# Response of Wheat DREB Transcription Factor to Osmotic Stress Based on DNA Methylation

**DOI:** 10.3390/ijms22147670

**Published:** 2021-07-18

**Authors:** Huihui Wang, Yanqiu Zhu, Ping Yuan, Shanglin Song, Tianyu Dong, Peilei Chen, Zhikun Duan, Lina Jiang, Longdou Lu, Hongying Duan

**Affiliations:** 1 College of Life Sciences, Henan Normal University, Xinxiang 453007, China; hui15690788798@163.com (H.W.); Qiqt536@163.com (Y.Z.); yp15249710089@163.com (P.Y.); dxdhy@163.com (S.S.); tianyuwithchen@163.com (T.D.); Chenpl536@163.com (P.C.); 041139@htu.edu.cn (L.J.); 2 College of Life Sciences, Henan University, Kaifeng 475004, China; duanzhikun@henu.edu.cn

**Keywords:** *Triticum aestivum*, osmotic stress, DREB transcription factor, promoter, DNA methylation

## Abstract

Dehydration-responsive element-binding protein (DREB) plays an important role in response to osmotic stress. In this study, *DREB2*, *DREB6* and *Wdreb2* are isolated from wheat AK58, yet they belong to different types of DREB transcription factors. Under osmotic stress, the transcript expression of *DREB2*, *DREB6* and *Wdreb2* has tissue specificity and is generally higher in leaves, but their expression trends are different along with the increase of osmotic stress. Furthermore, some elements related to stresses are found in their promoters, promoters of *DREB2* and *Wdreb2* are slightly methylated, but *DREB6*’s promoter is moderately methylated. Compared with the control, the level of promoter methylation in *Wdreb2* is significantly lower under osmotic stress and is also lower at CG site in *DREB2*, yet is significantly higher at CHG and CHH sites in *DREB2*, which is also found at a CHG site in *DREB6*. The status of promoter methylation in *DREB2*, *DREB6* and *Wdreb2* also undergoes significant changes under osmotic stress; further analysis showed that promoter methylation of *Wdreb2* is negatively correlated with their expression. Therefore, the results of this research suggest the different functions of *DREB2*, *DREB6* and *Wdreb2* in response to osmotic stress and demonstrate the effects of promoter methylation on the expression regulation of *Wdreb2*.

## 1. Introduction

Osmotic stress is one of the major abiotic stresses, which not only affects the growth and development of plants, but also severely restricts the sustainable production of agricultural biomass [1]. In order to adapt or resist to adverse environments, plants usually respond morphologically, physiologically and molecularly, such as by regulating the expression of stress resistance genes [2,3,4,5]. Plant resistances are generally controlled by multiple genes, it is well known that one transcription factor can regulate the expression of multiple functionally related genes, and many transcription factors are related to resistant response in plants, such as DREB, bZIP, MYB and WRKY [6].

DREB transcription factor belongs to AP2/EREBP family and contains one AP2/EREBP domain which is composed of about 60 amino acid residues with the conserved element YRG and RAYD [7]. Through the AP2/EREBP domain, DREB transcription factor could specifically bind to the dehydration responsive element/C-repeat (DRE/CRT) (core sequence: 5′-CCGAC-3′). This binding is involved in regulating the expression of genes related with abiotic stress response to high salt, low temperature or drought, which would enhance the resistances of plants to adverse stress [6]. *Arabidopsis* DREB1/CBF can regulate the expression of *rd29A*, *erd10*, *cor6.6*, *cor15a*, *rd17* and other stress-resistant genes to drought, low temperature and so on [8]. Overexpression of *DREB1A* in transgenic *Arabidopsis* also could enhance the expression of its downstream target genes, which increased the drought tolerance of transgenic *Arabidopsis* significantly [9]. So far, many genes encoding DREB transcription factor have been identified from *Arabidopsis*, *Maize*, *Soybean*, *Sesame*, etc.; their expression could be induced and would accumulate rapidly in a short time under abiotic stress [10]. However, the mechanisms of the regulation of *DREB* gene expression are less studied, especially the epigenetic regulations.

DNA methylation is a major epigenetic modification and has a vital function in the growth and development of plants [11]. DNA methylation in plants is easily affected by physiological status, developmental stage and environmental factors [12]. Under osmotic stress, the state of DNA methylation altered in plants, and methylation level and patterns were significantly different [13]. Fan et al. found that the methylation level of *Dendrobium huoshanense* decreased, and methylation polymorphism gradually increased along with the increase of osmotic stress [14]. The methylation level of *Ryegrass* also decreased under osmotic stress, and the expression of demethylated related genes was up-regulated [15]. Some studies indicated that the improved resistance of plants to stresses is connected with the involvement of DNA methylation in regulating the expression of stress-resistant genes [16]. For example, the physiological processes of *Rice* are related to DNA methylation in response to osmotic stress [13], the modification of DNA methylation status is closely connected with drought resistance of *Fraxinus* hybrid trees [17]. Furthermore, methylation or demethylation of genes in plants can cause the difference of gene expression under osmotic stress [18], which would result in drought escape or tolerance [19].

Wheat (*Triticum aestivum* L.), belonging to Gramineae family, is rich in starch, protein, sugar and other substances, and is one of the main food crops. In recent years, osmotic stress is a significant reason for the restriction of sustainable increase of wheat production [20]. However, studies on the response of wheat to osmotic stress are usually confined to phenotype, structure, physiology and biochemistry, or stress-resistant genes [21]. The epigenetic regulation of wheat response to osmotic stress is rarely involved, especially the regulatory role of DNA methylation in DREB transcription factor under osmotic stress. In this study, three members of the DREB family in wheat AK58 are identified, and the expression and promoter methylation of *DREB* genes are analyzed under osmotic stress. Thus, this investigation is beneficial to explore the regulatory mechanism of DNA methylation in the response of plants to osmotic stress.

## 2. Results

### 2.1. Amplification and Sequence Analysis of DREBs

As shown in Figure S1, CDS sequences of *DREB2*, *DREB6* and *Wdreb2* in wheat AK58 comprise 732 bp, 837 bp and 1035 bp respectively; *DREB2* and *Wdreb2* have no intron, but one 712-bp intron is found in *DREB6*. A typical AP2/EREBP domain is identified in the deduced protein sequences of three *DREB* genes (Figure S1). AP2/EREBP domain contains YRG and RAYD conserved modules with three β folds and one α helix, valine (V) and glutamate (E) are highly conserved at 14 th or 19 th residue of AP2/EREBP domain (Figure S2a). The amino acid sequences of DREB2, DREB6 and Wdreb2 are further compared and analyzed, and despite the low overall sequence similarity among three DREBs (33.24% identity) (Figure S2b), their AP2/EREBP domains have 73.25% identity, even up to 83.93% between DREB6 and Wdreb2 (Figure S2).

The deduced protein sequences of DREB2, DREB6 and Wdreb2 from wheat AK58 are also aligned with their homologous sequences (Figure S3). The sequence identity between DREB2 and *Aegilops tauschii* ERF is 95%, but has only around 60% identity with other homologous sequences (Table S1). As listed in Table S1, DREB6 has higher similarity to some homologous sequences (approximately 97% identity), the higher sequence similarity is observed between Wdreb2 and *Aegilops tauschii* DREB2B, *Aegilops speltoides* DREB1, *Triticum turgidum* DRF, *Triticum dicoccoides* DREB or *Triticum urartu* DREB2B (Table S1).

### 2.2. The Expression Patterns of DREBs in Wheat

**Figure 1:**
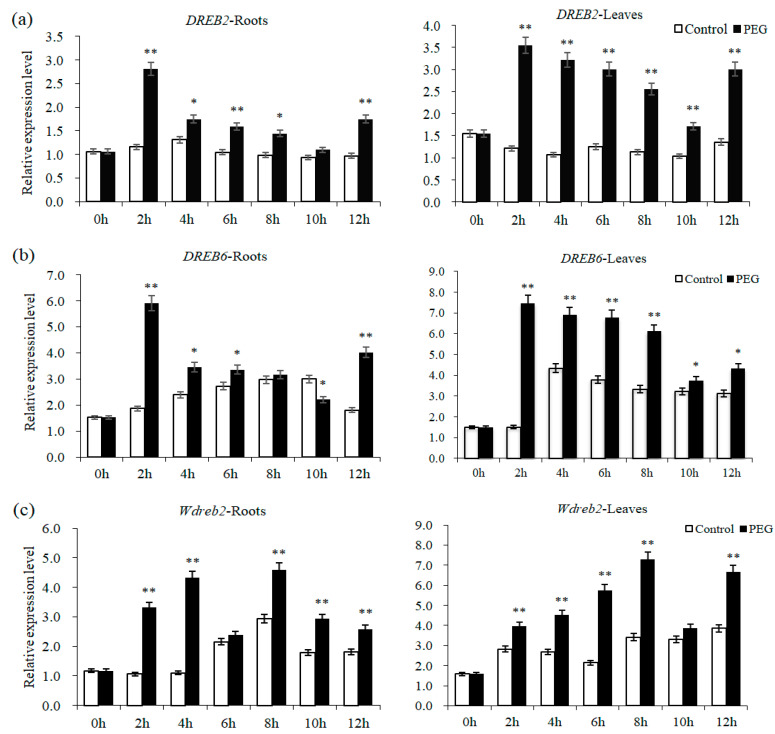
The expression analysis of wheat *DREB* genes under osmotic stress. Quantitative real-time PCR (qRT-PCR) analysis of *DREB2*, *DREB6* and *Wdreb2* expression are shown in (**a**–**c**); the transcript abundances of three *DREB*s are analyzed in wheat seedlings treated with 15% PEG6000 solution. The error bar is the standard error of the mean. * and ** represent significant difference relative to the control conditions at the levels of 0.05 and 0.01, respectively.

As shown in Figure 1a, the expression level of *DREB2* in leaves is significantly higher than that in roots under osmotic stress, similarly in *DREB6* as stressed for 2–10 h (Figure 1b). Compared with the level in roots, the expression level of *Wdreb2* in leaves is also significantly higher except for the stress of 4 h or 10 h (Figure 1c). Furthermore, the expression of *DREB2*, *DREB6* and *Wdreb2* is different under osmotic stress and has its own unique expression profile (Figure 1).

Under osmotic stress, the expression of *DREB2* displays similar trends in roots and leaves (Figure 1a). *DREB2* transcript abundance would increase to a higher level after stressed for 2 h (*p* < 0.01), then declines, and is lower after stressed for 10 h, which is still higher in roots and leaves than the control and is especially significant in leaves (*p <* 0.01). The expression trends of *DREB6* are also similar in roots and leaves under osmotic stress (Figure 1b). A significant rise in the *DREB6* transcript level is also observed after stressed for 2 h (*p* < 0.01), although *DREB6* subsequently exhibits the declined expression, which is significantly higher in leaves than the control (Figure 1b). As shown in Figure 1c, under osmotic stress, *Wdreb2* is up-regulated in roots except for the stress of 6 h, especially after stressed for 2–4 h (*p* < 0.01). Compared with the control, except for the expression stressed for 10 h, *Wdreb2* transcript level in leaves also increases significantly under osmotic stress, especially after stressed for 6–8 h (*p* < 0.01).

### 2.3. Promoter Analysis of Wheat DREB Genes

In this study, the promoters of *DREB2*, *DREB6* and *Wdreb2* are isolated and submitted to GenBank (MT974473: 1735 bp, MT974471: 1792 bp, MT974472: 649 bp). As shown in Figure S4 and Tables S2–S4, the promoters of wheat *DREB* genes contain basic regulatory elements, such as TATA-box and CAAT-box. There are 13, 10 and 5 TATA-boxes in the promoters of *DREB2*, *DREB6* and *Wdreb2*, respectively. Many elements related to stresses are also found in the promoters of *DREB2*, *DREB6* and *Wdreb2* such as drought response element DRE/CRT, low temperature response element LTR, abscisic acid response element ABRE, light response element GAG-motif, drought-induced element MYB binding sites, etc. (Figure S4, Tables S2–S4).

Further analysis showed that there are some unique elements in the promoters of *DREB2*, *DREB6* and *Wdreb2*. For example, light response element MNF, leaf development element HD-ZIP and meristem specificity element OCT are specifically present in the promoter of *DREB2* (Table S2). A series of specific functional elements are also identified in the promoter of *DREB6*, such as ethylene response element ERE, fungal elicitor response element W-box and MeJA regulatory element CGTCA-motif (Table S3). Moreover, root specificity elements as1, zein metabolism regulation element O2-site, light response element C-box, and CE3 element involved in ABA and VP1 reactions are detected in the promoter of *Wdreb2* (Table S4).

### 2.4. Promoter Methylation Analysis of DREB Genes

The distribution of CpG island in the promoter regions of whet *DREBs* are analyzed. It is found that one CpG island is present in the promoter of *DREB2*, *DREB6* or *Wdreb2*, and its length is 234 bp, 436 bp and 559 bp, respectively (Figure S5). These putative CpG islands are preceded by some functional elements such as the abscisic acid responsive element, light responsive element, low-temperature responsive element and so on (Figure S4, Tables S2–S4).

Some CpG island regions with a higher CG percentage are further identified in wheat leaves (Figure S6). The majority of methylation sites are in CHH context in the promoter regions of *DREB2*, *DREB6* and *Wdreb2*, but DNA methylation has a strong preference for CG context (Table 1). In the promoter region of *DREB2*, methylation in CHH site is not detected and methylation rates of CG and CHH sites are 2.38% and 1.03%, belonging to mild methylation (Figure S6a; Table 1). Figure S6b and Table 1 shows dense methylation at CG site (88.08%), moderate methylation at CHG site (51.36%) and mild methylation at CHH site (4.93%) in the promoter region of *DREB6*. In the promoter region of *Wdreb2*, methylation rates of CG, CHG and CHH sites are 1.89%, 1.0% and 0.29%, respectively, which are all mildly methylated (Figure S6c; Table 1).

### 2.5. Methylation Level of DREB Promoters under Osmotic Stress

**Figure 2:**
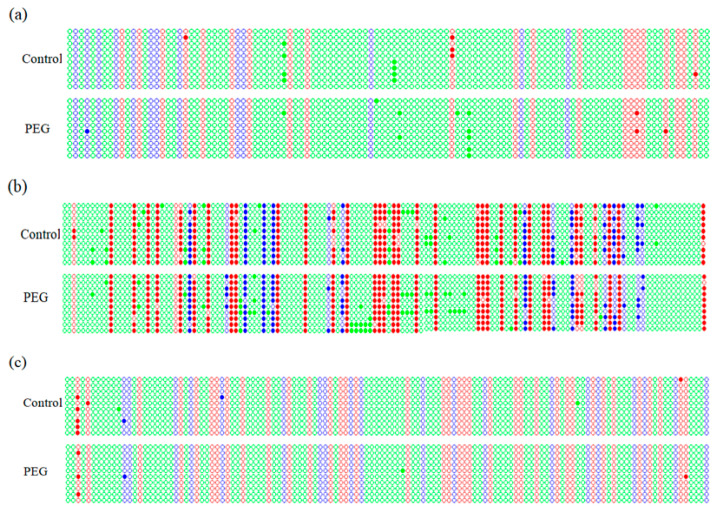
Methylation profiles in the promoter regions of wheat *DREB* genes under osmotic stress. (**a**–**c**) represent cytosine methylation maps for the promoter sequences of *DREB2*, *DREB6* and W*dreb2* in wheat leaves stressed with 15% PEG6000 solution for 12 h. Red, blue and green circles represent CG, CHG or CHH. Filled and hollow circles denote methylated and unmethylated cytosine, respectively. Each row represents the sequencing result of one positive clone.

**Figure 3:**
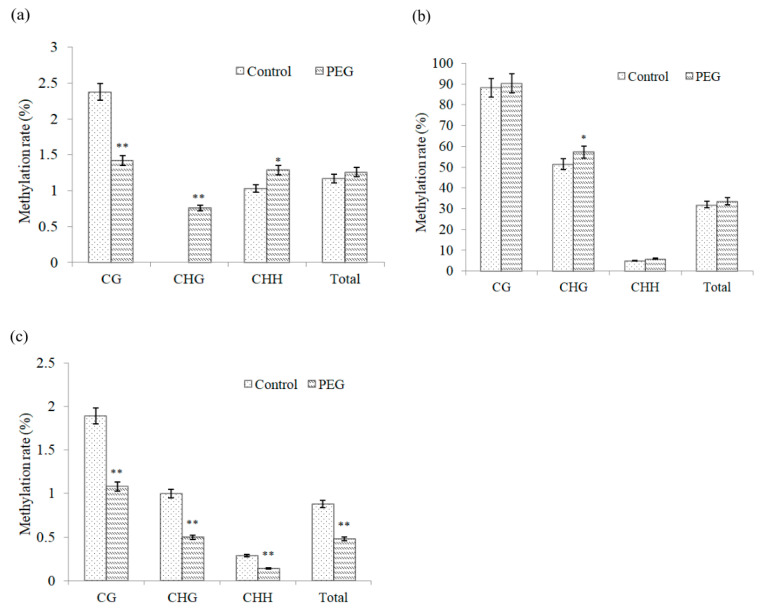
Effect of osmotic stress on methylation level of *DREB* promoters in wheat. (**a**–**c**) represent methylation rates for the promoter regions of *DREB2*, *DREB6* and W*dreb2* in wheat leaves stressed with 15% PEG6000 solution for 12 h. The error bar is the standard error of the mean. * and ** represent significant difference relative to the control conditions at the levels of 0.05 and 0.01, respectively.

Under osmotic stress, cytosine methylation would alter in the promoter regions of *DREB2*, *DREB6* and *Wdreb2* from wheat leaves (Figure 2). Compared with the control, methylation rate at CG site in the promoter region of *DREB2* decreases obviously under osmotic stress (*p* < 0.01), but methylation rates at CHG and CHH sites increase significantly, especially in CHG site (*p* < 0.01) (Figure 3a).

As shown in Figure 3b, under osmotic stress, the increased methylation level of *DREB6* promoter is observed, which only occurrs at CHG site (*p* < 0.05). Figure 3c displays the changes in promoter methylation of *Wdreb2* under osmotic stress. Compared with the control, the overall methylation level was significantly lower (*p* < 0.01); methylation rates at CG, CHG and CHH sites also significantly declines under osmotic stress (*p* < 0.01).

### 2.6. Methylation Status in DREB Promoters under Osmotic Stress

As listed in Table 2, methylation status in the promoter regions of *DREB2*, *DREB6* and *Wdreb2* alters significantly under osmotic stress for 12 h. The demethylation at 3 CG sites and 1 CHH site are detected in the promoter of *DREB2*, and the hypermethylation of *DREB2* promoter at CG, CHG and CHH sites is also found, being 2, 1 and 3, respectively.

Under osmotic stress, 10 CHH sites, 1 CG site and 1 CHG site are hypermethylated in the promoter of *DREB6*. 8 CHH sites, 1 CG site and 1 CHG site are demethylated (Table 2). Compared to CHG sites, the methylation status of CHH and CG sites in *Wdreb2* promoter are affected strongly by osmotic stress (Table 2): for example, 1 CHH site and 1 CG site are hypermethylated, whereas 2 CHH sites and 2 CG sites are demethylated.

### 2.7. Correlation Analysis between Promoter Methylation and Expression of DREBs

In order to explore the correlation between promoter methylation and expression of wheat *DREBs* under osmotic stress, the relative expression levels of *DREB*s in wheat leaves and methylation rates at CG, CHG or CHH sites in their promoter regions were respectively analyzed by Pearson correlation coefficient. As listed in Table S5, Pearson coefficient r between the expression level of *Wdreb2* and methylation rate at CG, CHG and CHH sites is respectively −0.986, −0.973 and −0.878, indicating that a significant negative correlation exists between promoter methylation and the gene expression of *Wdreb2*. Although significant negative correlation exists between the gene expression of *DREB2* or *DREB6* and the methylation rate of CG (Table S5), promoter methylation of *DREB2* or *DREB6* has no negative correlation with its expression under osmotic stress.

## 3. Discussion

DREB transcription factor can specifically bind to DRE/CRT elements in the promoter of stress-responsive genes and can enhance the response or tolerance of plants to adverse environmental conditions [6]. In this study, *DREB2*, *DREB6* and *Wdreb2* are isolated from wheat AK58, and one 712-bp intron is found in *DREB6*, AP2/EREBP domains of DREB2, DREB6 and Wdreb2 have 73.25% identity; the amino acids at the 14th and 19th of AP2/EREBP domain are conservatively V and E, respectively. However, the similarity is lower among the full-length nucleotide sequences or amino acid sequences of *DREB2*, *DREB6* and *Wdreb2*. BLASTP analysis further revealed that DREB2, DREB6 and Wdreb2 are different types of DREB transcription factors and might respectively belong to DREBA-4 class, DREB-2 class and DREB-1 class [22,23].

Under abiotic stresses such as drought, low temperature, high salt, etc., the expression of DREB transcription factor can change [24,25]. In this study, the expression of *DREB2*, *DREB6* and *Wdreb2* also alters under osmotic stress and generally accumulates to the higher level after stressed for 2 h, but they have different expression trends along with the increase of stress time. The expression levels of wheat *DREBs* are also different, which is similar to other research [26]. Further analysis showed that the expression of wheat *DREBs* has tissue specificity, and the accumulation of *DREBs* transcripts is obviously higher in leaves, which is also found in other research [27]. The expression of *DREB* in *Daucus carota* showed tissue specificity as well, and the main role of *DcDREB-A1-1* and *DcDREB-A1-2* is in leaves and roots, respectively [28].

It is well known that the *cis*-acting regulatory elements in the promoters provide the possibility for the transcription and expression of genes [29], and there are some *cis*-acting elements related to stresses, such as DRE/CRT, ERE, ABRE, LTR and so on [30]. Except typical regulatory elements TATA-box and CAAT-box, the promoters of *DREB2*, *DREB6* and *Wdreb2* in wheat AK58 contain DRE/CRT, LTR, ABRE, drought-induced MYB binding site, etc., which demonstrates that the expression of *DREB2*, *DREB6* and *Wdreb2* may be influenced by adverse environmental factors. Some studies found that DNA methylation can regulate the expression of stress-responsive genes and play an important role in the response of plants to adverse stress [16], especially promoter methylation which has more significant effects on gene expression [31]. In the promoter regions of *DREB2*, *DREB6* and *Wdreb2*, CpG islands with many *cis*-acting elements are detected. BSP analysis showed that there are more CHH sites and less CHG sites in the promoter regions of wheat *DREBs*, but the methylation rates at CG sites are the highest.

Furthermore, the degree and state of DNA methylation in plants can change under drought stress, low temperature, high salt and other conditions [32,33], especially the change of methylation state in the promoter of some genes [34]. In this study, methylation level also alters in the promoter regions of *DREBs* under osmotic stress. Compared with the control, the level of promoter methylation in *Wdreb2* is significantly lower under osmotic stress and is also lower at the CG site in *DREB2*, yet is significantly higher at CHG and CHH sites in *DREB2*, which is also found at the CHG site in *DREB6*. In addition, methylation status in the promoter regions of wheat *DREBs* undergoes a significant change under osmotic stress, such as demethylation and hypermethylation. Zilberman also found that gene expression can be promoted or inhibited by the demethylation and hypermethylation of promoter, respectively [35].

Further analysis showed that promoter methylation of *Wdreb2* is negatively correlated with their expression, which is also found in other studies [35,36]. Although the promoters of *DREB2* and *Wdreb2* are both slightly methylated, the expression of *Wdreb2* was significantly higher than that of *DREB2*, indicating that promoter methylation might have little effect on the gene expression of *DREB2* and its promoter possibly belongs to a low CpG-containing promoter. Similarly, the promoters of *z1B4* and *z1B6* in *Zea mays* are almost not methylated [37]; DNA methylation is also not found in the promoters of some genes in *Arabidopsis* or tomato and only occurs in their coding regions [38,39]. In addition, one CpG island is also found in the coding region of wheat *DREBs*, and the CpG island almost covers the whole coding region of *DREB2*. However, the relationship between DNA methylation in the coding region and gene expression of wheat *DREB* genes is unclear; the mechanism of DNA methylation regulating the expression of wheat *DREB* genes therefore needs to be further studied.

## 4. Materials and Methods

### 4.1. Cultivation and Treatment of Wheat Seedlings

In this study, seeds of wheat AK58 are kindly provided by Xinxiang Academy of Agricultural Science, Xinxiang, Henan, China. Cultivation of wheat seedlings is performed according to Duan et al. [33], wheat seeds are firstly surface-sterilized for 10 min by 0.1% HgCl_2_ and then are washed for 50 min in sterile water. Subsequently, sterilized seeds are sown in pots (with a diameter of 15 cm) containing nutrition soil and vermiculite (1:1), cultured at 24 ± 1 °C with 45% relative humidity in a 14 h photoperiod of 50 μmol m^−2^ s^−1^ light intensity, and finally are irrigated with 5 mL distilled water every two days.

At the three-leaf stage, wheat seedlings are exposed to 15% PEG6000 solution for 2 h, 4 h, 6 h, 8 h, 10 h and 12 h. Roots and leaves of PEG6000-treated and untreated wheat seedlings are collected, immediately frozen with liquid nitrogen and then stored at −80 °C.

### 4.2. Extraction of Genomic DNA

Genomic DNA is extracted from roots or leaves of wheat seedlings by the cetyltriethyl ammonium bromide (CTAB) method [40]. The yield and purity of genomic DNA are determined at 260 nm with micro-spectrophotometry, and the integrity of genomic DNA is detected by 0.8% agarose gel electrophoresis. Subsequently, genomic DNA from wheat seedlings is stored at −20 °C.

### 4.3. Isolation and Reverse Transcription of RNA

Total RNA from roots or leaves of wheat seedlings is extracted using RNAiso Plus kit (TaKaRa, Kyoto, Japan) according to the instructions. DNase treatment and phenol-chloroform extraction are performed to remove DNA, and RNA samples are dissolved in RNase-free ddH_2_O. The integrity of total RNA is verified by 1.0% agarose gel electrophoresis, and the yield and purity of total RNA are determined with UV spectrophotometer. RNA samples are used for the generation of cDNA or stored at −80 °C. First strand cDNA is synthesized with the HiScript II Q RT SuperMix for qPCR (+gDNA wiper) kit (Vazyme, Nanjing, China).

### 4.4. Amplification and Bioinformatic Analysis of DREB Genes

In order to isolate *DREB* genes from wheat AK58, specific primers are designed according to the sequences of wheat *DREB2* (GU785008), *DREB6* (AY781361) and *Wdreb2* (AB193608), and are listed in Table S6. Genomic DNA and cDNA of wheat AK58 were used as the amplification templates to obtain DNA or cDNA sequences of *DREB* genes.

In this experiment, a PCR reaction system is composed of a 2.0 μL DNA template, 1.0 μL for each primer (10 μM), 10.0 μL 2× Taq Mix and 6.0 μL ddH_2_O. The following PCR procedure is used for the amplification: 95 °C for 5 min, followed by 35 cycles (94 °C for 30 s, 55 °C for 30 s and 72 °C for 1 min), final extension at 72 °C for 5 min. After PCR amplification products are detected by 1.0% agarose gel electrophoresis, target fragments are obtained by gel extraction and recycling and then are sequenced in Vazyme (Nanjing, China).

Bioinformatic analysis of target sequences is performed as described below: extrons, introns and ORFs of wheat *DREB* genes are analyzed with ProtParam (Expasy, Swiss), the conserved domains of amino acid sequences encoded by wheat *DREB* genes are analyzed by CD-search (NCBI, Bethesda, MD, USA), these homologous sequences are retrieved with BLASTP 2.12.2.0 (http://blast.ncbi.nlm.nih.gov/Blast.cgi, accessed on 30 June 2021) (NCBI, Bethesda, MD, USA), and the alignments on amino acid sequences or homologous sequences of wheat DREBs are carried out using DNAMAN 6.0 (Lynnon Biosoft, San Ramon, CA, USA).

### 4.5. Fluorescence Quantitative Real-Time PCR

The expression of *DREB* genes in wheat is studied by qRT-PCR, the internal reference gene is *β-Actin*, and these primers for qRT-PCR are listed in Table S6. qRT-PCR is performed on LightCycler 96 Real-time PCR instrument, and cDNA synthesized by reverse transcription of total RNA is used as the template in qRT-PCR.

According to the instructions of AceQ qPCR SYBR Green Master Mix kit (Vazyme, Nanjing, China), qRT-PCR reaction system consists of 1.0 μL AceQ qPCR SYBR Green Master Mix, 0.5 μL for each primer (10 μM), 2.0 μL cDNA template and 16.0 μL ddH_2_O. qRT-PCR is performed with the following procedures: pre-denaturation for 5 min at 95 °C, followed by 40 cycles of 95 °C for 10 s and 60 °C for 30 s.

The relative expression level of wheat *DREB* genes under osmotic stress is normalized and analyzed by the comparative Ct (^2−ΔΔct^) method [41]. The calculation formula is as follows: Relative expression level =2^−∆∆Ct^, ∆∆Ct (target gene) =∆Ct (treatment group) − ∆Ct (control group), ∆Ct (target gene) =Ct (target gene) − Ct (reference gene). Furthermore, data are obtained from three biological replicates, and each qRT-PCR experiment is repeated three times.

### 4.6. Isolation and Analysis of Promoter Sequence

The promoter regions are isolated to further analyze the expression pattern of *DREB* genes in wheat AK58. Specific primers are designed according to the promoter sequences of wheat *DREB2* (GU785008), *DREB6* (HG670306.1) or *Wdreb2* (KF731666), and are listed in Table S6.

The target promoter sequences are amplified in 20 μL PCR reaction mixture consisting of 2.0 μL DNA template, 10.0 μL 2× Taq Mix, 1.0 μL for each primer (10 μM) and 6.0 μL ddH_2_O. The reaction condition of PCR procedure is 95 °C for 5 min, followed by 40 cycles (94 °C for 30 s, 55 °C for 30 s and 72 °C for 1.5 min), with the final extension at 72 °C for 5 min. PCR amplification products are separated by 1.0% agarose gel electrophoresis. The target fragments are obtained by gel extraction and recycling, then are sequenced in Vazyme (Nanjing, China). PlantCAREand PLACE are used to analyze *cis*-acting elements in the promoter sequences of wheat *DREB* genes.

### 4.7. Methylation Analysis of Promoter

CpG islands (Island size > 100, GC Percent > 50.0, Obs/Exp > 0.6) in the promoter regions of wheat *DREBs* are predicted and analyzed using EMBOSS CpG Plot (European Bioinformatics Institute, Cambridge, UK). According to the analysis of CpG islands, amplification primers for bisulfite sequencing PCR (BSP) are designed using MethPrimer (Chinese Academy of Medical Sciences, Beijing, China), Methyl Primer Expressv 1.0 (Applied Biosystems, Foster, CA, USA) and Primer Premier 5.0 (Premier Biosoft International, Palo Alto, Canada), and are listed in Table S5. The CpG island of *DREB6* promoter is amplified in two parts (region I and region II) because of the limited length of BSP amplification.

In this study, genomic DNA from wheat leaves is firstly treated with EZ DNA Methylation-Lightning^TM^ Kit (Zymo Research, Irvine, CA, USA), and then used as the template in BSP amplification of *DREB* promoters. BSP reaction system is 30 μL and composed of 2.0 μL bisulfite-treated DNA, 1.0 μL for each primer (10 μM), 3.0 μL 10× buffer (Mg^2+^), 1.0 μL dNTP, 1.0 μL Relia™ hot-start Taq polymerized aes and 21.0 μL ddH_2_O. The PCR amplification procedure is as follows: pre-denaturation at 95 °C for 4 min, followed by 40 cycles (94 °C for 30 s, 55 °C for 30 s, 72 °C for 40 s), and a final extension at 72 °C for 5 min. PCR amplification products are detected by 1.0% agarose gel electrophoresis. It is found that only target fragments are amplificated. The target fragments are obtained by gel extraction and recycling, and are sequenced by GENEray (Shanghai, China).

In addition, at least 10 clones are sequenced for each target fragment and three biological replicates are set up; the analysis on methylation site, methylation type and methylation rate are performed with CyMATE (Vienna University of Technology, Vienna, Austria) and Kismeth (Mount Sinai School of Medicine, New York, USA).

### 4.8. Statistical Analysis

Statistical analysis of data is performed in this study; the expression level of genes and methylation ratio of promoters are tested by significance level, ANOVA and multiple comparisons of Duncan’s multiple range; the correlation between gene expression and promoter methylation is analyzed by Pearson correlation coefficient r of SPSS 15.0 (SPSS, New York, USA).

## 5. Conclusions

In this study, *DREB2*, *DREB6* and *Wdreb2* are identified in wheat AK58, and one 712-bp intron is found in *DREB6*. Under osmotic stress, the expression patterns of *DREB2*, *DREB6* and *Wdreb2* are different, yet their expression levels are obviously higher in leaves. Some elements related to stresses are also found in the promoter regions of *DREB2*, *DREB6* and *Wdreb2*, while further analysis showed that promoter methylation of *Wdreb2* is negatively correlated with their expression. Therefore, *DREB2*, *DREB6* and *Wdreb2* in wheat might function differently in response to osmotic stress, and promoter methylation has more significant effects on the gene expression of *Wdreb2*, which would be helpful to reveal the regulatory mechanism of DNA methylation in plant response to osmotic stress.

## Figures and Tables

**Table 1:** Methylation analysis of promoter regions in wheat *DREB* genes.

Gene	Pattern	Pattern Frequency (%)	Methylation Rate (%)	Total Methylation Rate (%)
*DREB2*	CG	19.09	2.38	1.17
	CHG	11.82	0.00	
	CHH	69.09	1.03	
*DREB6*	CG	25.93	88.08	31.89
	CHG	11.11	51.36	
	CHH	62.96	4.93	
*Wdreb2*	CG	29.60	1.89	0.88
	CHG	16.00	1.00	
	CHH	54.40	0.29	

**Table 2:** Methylation patterns in promoter regions of wheat *DREB*s under osmotic stress.

Gene	Cytosine Type	No. of Cytosine	No. of Methylation Site	
			Hypermethylation Site	Demethylation Site
*DREB2*	CG	21	2	3
	CHG	13	1	0
	CHH	76	3	1
*DREB6*	CG	34	1	1
	CHG	15	1	1
	CHH	84	10	8
*Wdreb2*	CG	37	1	2
	CHG	20	0	1
	CHH	68	1	2

## Data Availability

The data presented in the current study are available in the article and supplementary materials.

## Supplementary Materials

The following are available online at https://www.mdpi.com/article/10.3390/ijms22147670/s1. Figure S1 The sequences of wheat *DREB* genes. (**a**), (**b**) and (**c**) represent the nucleotide sequence and amino acid sequence of *DREB2*, *DREB6* and *Wdreb2*, respectively. The box and underline denote nuclear localization signal and AP2/EREBP domain, respectively. Figure S2 Alignments of wheat DREBs. (**a**) and (**b**) represent the alignments of AP2/EREBP domain and amino acid sequence of *DREB2*, *DREB6* and *Wdreb2*. The similar and consistent amino acids are indicated in blue or dark blue, respectively. β-sheet and α-helix are indicated with long arrow and dotted line, and the conserved residues at 14th and 19th are marked with short arrow. Figure S3 Multiple alignment of wheat DREBs and its homologous sequences. (**a**), (**b**) and (**c**) are the alignment on some homologous sequences of wheat DREB2, DREB6 and Wdreb2, respectively. The underline denotes the AP2/EREBP domain. Figure S4 Promoter regions of wheat *DREB* genes. (**a**), (**b**) and (**c**) represent the promoter sequences and some *cis*-acting elements of *DREB2*, *DREB6* and *Wdreb2*, respectively. Figure S5 Prediction of CpG islands in the promoter regions of wheat *DREB* genes. (**a**), (**b**) and (**c**) represent the location of putative CpG islands in the promoter regions of *DREB2*, *DREB6* and *Wdreb2*, respectively. The CpG island regions between two arrows below each diagram were examined using BSP. Figure S6 Bisulfite sequencing analysis on the promoter regions of wheat *DREB* genes using CyMATE. (**a**), (**b**) and (**c**) indicate methylation sites in the promoter regions of *DREB2*, *DREB6* and W*dreb2* in wheat leaves. Circles, squares and triangles represent CG, CHG or CHH; filled and hollow circles denote methylated and unmethylated cytosine. Each row represents the sequencing result of one positive clone. The length of CpG island sequence is visualized above each diagram and the potential methylation sites are displayed below. Table S1 Homologous amino acid sequences of wheat DREBs by BLASP. The homologous sequences of wheat DREB2, DREB6 and Wdreb2 were retrieved with BlastP algorithm, which are mainly from some species in Gramineae. The homologous sequences having higher identity with wheat DREBs were selected from some Genera in Gramineae and were further analyzed. Table S2 The *cis*-acting elements in the promoter of *DREB2.* Table S3 The *cis*-acting elements in the promoter of *DREB6*. Table S4 The *cis*-acting elements in the promoter of *Wdreb2*. Table S5 Correlation between promoter methylation and expression of *DREB* genes. Pearson’s r represents the relationship between expression levels of *DREB2, DREB6, Wdreb2* in wheat leaves and methylation rates at CG, CHG or CHH sites in their promoter regions under the treatment of 15% PEG_6000_ solution. According to the expression level and methylation rate of wheat seedlings incubated in PEG_6000_ solution for 12 h, Pearson’s r analysis of *DREBs* were performed. Table S6 Primers used in this study.

## Abbreviations

BSP	bisulfite sequencing PCR
CTAB	cetyltriethyl ammonium bromide
DRE/CRT	dehydration responsive element/C-repeat
DREB	dehydration responsive element binding protein
qRT-PCR	quantitative real-time PCR
