# Supplementary material for: Response of Wheat DREB Transcription Factor to Osmotic Stress Based on DNA Methylation
Source: Int J Mol Sci. 2021 Jul 18;22(14):7670. doi: 10.3390/ijms22147670 (PMC8307787; doi:10.3390/ijms22147670)
Supplement: Supplementary file 1 [file ijms-22-07670-s001.zip › ijms-1300145-supplementary.pdf]

Supporting information

Figure S1 The sequences of wheat *DREB* genes. (a), (b) and (c) represent the nucleotide sequence and amino acid sequence of *DREB2*, *DREB6* and *Wdreb2*, respectively. The box and underline denote nuclear localization signal and AP2/EREBP domain, respectively.

Figure S2 Alignments of wheat DREBs. (a) and (b) represent the alignments of AP2/EREBP domain and amino acid sequence of *DREB2*, *DREB6* and *Wdreb2*. The similar and consistent amino acids are indicated in blue or dark blue, respectively. β-sheet and α-helix are indicated with long arrow and dotted line, the conserved residues at 14 th and 19 th are marked with short arrow.

Figure S3 Multiple alignment of wheat DREBs and its homologous sequences. (a), (b) and (c) are the alignment on some homologous sequences of wheat DREB2, DREB6 and Wdreb2, respectively. The underline denotes the AP2/EREBP domain.

Figure S4 Promoter regions of wheat *DREB* genes. (a), (b) and (c) represent the promoter sequences and some *cis*-acting elements of *DREB2*, *DREB6* and *Wdreb2,* respectively.

Figure S5 Prediction of CpG islands in the promoter regions of wheat *DREB* genes. (a), (b) and (c) represent the location of putative CpG islands in the promoter regions of *DREB2*, *DREB6* and *Wdreb2*, respectively. The CpG island regions between two arrows below each diagram were examined using BSP, respectively.

Figure S6 Bisulfite sequencing analysis on the promoter regions of wheat *DREB* genes using CyMATE. (a), (b) and (c) indicate methylation sites in the promoter regions of *DREB2*, *DREB6* and W*dreb2* in wheat leaves. Circles, squares and triangles represent CG, CHG or CHH, filled and hollow circles denote methylated and unmethylated cytosine. Each row represents the sequencing result of one positive clone. The length of CpG island sequence is visualized above each diagram and the potential methylation sites are displayed below.

Table S1 Homologous amino acid sequences of wheat DREBs by BLASP

The homologous sequences of wheat DREB2, DREB6 and Wdreb2 were retrieved with BlastP algorithm, which are mainly from some species in Gramineae. The homologous sequences having higher identity with wheat DREBs were selected from some Genera in Gramineae, and were further analyzed.

Table S2 The *cis*-acting elements in the promoter of *DREB2*

Table S3 The *cis*-acting elements in the promoter of *DREB6*

Table S4 The *cis*-acting elements in the promoter of *Wdreb2*

Table S5 Correlation between promoter methylation and expression of *DREB* genes. Pearson's r represents the relationship between expression levels of *DREB2, DREB6, Wdreb2* in wheat leaves and methylation rates at CG, CHG or CHH sites in their promoter regions under the treatment of 15% PEG_{6000} solution. According to the expression level and methylation rate when wheat seedlings incubated in PEG_{6000} solution for 12 h, Pearson's r analysis of *DREBs* were performed.

Table S6 Primers used in this study

(a)

```
1     CTACTCCCTGCACGGTCCTCCCATTACCACGAGCGAGCGCGTGTCATCTCATACGGGCACGCGGGGCTACGTGGT
76    GCTCTCTGCGCGCACATGGCGGCGAGCGAGCAGAGCTCCGAGTCCTCGTCGACGTCCTGCACTTCCTCCTACTCC
1                         M  A  A  S  E  Q  S  S  E  S  S  S  T  S  C  T  S  S  Y  S
151   ACGTCCTCGTGCTCCCCCAGGGCCGACGACAAGAAGCGGAACGGCGGCGGCAAGCGGAAGCGCGCAGCGGCGGCC
21     T  S  S  C  S  P  R  A  D  D  K  K  R  N  G  G  G  K  R  K  R  A  A  A  A
226   GACGAGGAGCCGGCCCCGGCGGCGGCGGCGGCGTCGTATCGCGGCGTGCGGATGCGGGCGTGGGGCAAGTGGGTG
46     D  E  E  P  A  P  A  A  A  A  A  S  Y  R  G  V  R  M  R  A  W  G  K  W  V
301   TCGGAGATCAGGGAGCCGCGCAAGAAGTCCCGCATCTGGCTCGGCACCTTCCCGTGCCCGGAGATGGCCGCGCGC
71     S  E  I  R  E  P  R  K  K  S  R  I  W  L  G  T  F  P  C  P  E  M  A  A  R
376   GCCCACGACGCCGCCGCGCTCAGCATCAAGGGCGCCCGCGCCGTGCTTAACTTCCCGGACCTCGCGCCCGCGCTC
96     A  H  D  A  A  A  L  S  I  K  G  A  R  A  V  L  N  F  P  D  L  A  P  A  L
451   CCTCGCCCGGCCTCCCTCGCCCCGTGCGACGTCCAGGCCGCCGCCGCGCGCGCCGCCCTCATGCACGACCACGAC
121    P  R  P  A  S  L  A  P  C  D  V  Q  A  A  A  A  R  A  A  L  M  H  D  H  D
526   CACCAGTGCTGCTCCGCCTCAACCTCCCCCGCCGCCGCGCCGGACTCCGCGCGCGGCAATGCCCCGGCGCCATGC
146    H  Q  C  C  S  A  S  T  S  P  A  A  A  P  D  S  A  R  G  N  A  P  A  P  C
601   GACCAGCCGGCCGCGCGAGACGAGCCGGAGCATGAGCCGCCGTTCCAGCCGAGCCAGGGAAATGAGCAGCAGCTC
171    D  Q  P  A  A  R  D  E  P  E  H  E  P  P  F  Q  P  S  Q  G  N  E  Q  Q  L
676   ACGGCTGCACAGGTGGAGATGGTGTTCGACGAGCTGGCGCCGCTGTGGGTGGAGAACGTGGTGGATTTCGCGCCG
196    T  A  A  Q  V  E  M  V  F  D  E  L  A  P  L  W  V  E  N  V  V  D  F  A  P
751   TCCGATCACTGCTGGACGGCGTACGATTGTCTCGACCCAATCGGCTTCCAGCCTCTCCTGTGGGAGTATTAGGCT
221    S  D  H  C  W  T  A  Y  D  C  L  D  P  I  G  F  Q  P  L  L  W  E  Y
```

(b)

```
1     TCGTCCCTCTTCTCGCTCCATGGAGACCGGGGGTAGCAAGCGGGAAGGAGACTGCCCCGGGCAGGAAAGgtgagc
1                           M  E  T  G  G  S  K  R  E  G  D  C  P  G  Q  E  R
76    gccggaaccactccctccatctctacttgtatcccgccgcgtttcgctcgaatctacgggcattgctgaggcggc
151   ggcggcgcgccgtcgccgcgacatggaatttgcgcgtgtttccgcggttggtcggtgctgtgaaacatgcccatc
226   taaagcagttatccgctactcgtttcctgttaccgcggcgcgtgtcggactagtcgtaggtcggcggtggtcgcc
301   ctaggtcgatggctgtcctccagttcggttcgccgggtttccgccggctgtagaattatggtttcctccgtcttc
376   tcggttggggattggagttcggcgagattcagagcggtgcgtggcgaactgtcggtgttgggtcgggtgaggcgg
451   cgccgtcctctgaatcggatgcagaagtccccatgtccatggcggcggtcggggattgcccatgccatggcggta
526   cgattgctggtgcacagcatcataaactatatcttcgggagctcgattgcggttggtacccaacccaagtgataa
601   taatctccttgactttttccaccaaggaaacaaggatagccctgcttcgttttgttttagatttatacgacttt
676   ttttttctgtgagaaagattcatatgactctgactgcttatgtttttggtttcaacgtgttttcaccttgtgata
751   tggattgccttgatgaacagGAAGAAGAAAGTGCGCAGGAGAAGCACTGGTCCTGATTCGGTTGCTGAAACCATC
1                           K  K  K  V  R  R  R  S  T  G  P  D  S  V  A  E  T  I
826   AAGAAGTGGAAGGAGGAAAACCAGAAGCTCCAGCAAGAGAATGGATCCCGGAAAGCACCGGCCAAGGGTTCCAAG
36     K  K  W  K  E  E  N  Q  K  L  Q  Q  E  N  G  S  R  K  A  P  A  K  G  S  K
901   AAAGGGTGCATGGCAGGGAAAGGAGGTCCAGAGAATTCAAACTGCGCTTACCGCGGTGTGAGGCAGAGGACGTGG
61     K  G  C  M  A  G  K  G  G  P  E  N  S  N  C  A  Y  R  G  V  R  Q  R  T  W
976   GGGAAATGGGTTGCTGAGATCCGTGAGCCCAACCGTGGCAATCGGCTGTGGCTTGGTTCATTCCCTACCGCAGTC
86     G  K  W  V  A  E  I  R  E  P  N  R  G  N  R  L  W  L  G  S  F  P  T  A  V
1051  GAAGCTGCACGTGCATATGATGATGCGGCAAGGGCAATGTATGGCGCCAAAGCACGTGTCAACTTCTCAGAGCAG
111    E  A  A  R  A  Y  D  D  A  A  R  A  M  Y  G  A  K  A  R  V  N  F  S  E  Q
1126  TCCCCGGATGCCAACTCTGGTTGCACGCTGGCACCTCCATTGCCGATGTCTAATGGGGCAACCGCTGCGTCACAT
136    S  P  D  A  N  S  G  C  T  L  A  P  P  L  P  M  S  N  G  A  T  A  A  S  H
1201  CCTTCTGATGGGAAGGATGAATCGGAGTCTCCTCCTTCTCTTATCTCAAATGCGCCGACAGCTGCGCTGCATCGG
161    P  S  D  G  K  D  E  S  E  S  P  P  S  L  I  S  N  A  P  T  A  A  L  H  R
1276  TCTGATGCTAAGGATGAGTCTGAGTCTGCAGGGACCGTGGCACGTAAGGTGAAAAAAGAAGTGAGCAATGATTTG
186    S  D  A  K  D  E  S  E  S  A  G  T  V  A  R  K  V  K  K  E  V  S  N  D  L
1351  AGAAGTACCCATGAGGAGCACAAGACCCTGGAAGTATCCCAACCAAAAGGGAAGGCTTTACATAAAGCAGCGAAC
211    R  S  T  H  E  E  H  K  T  L  E  V  S  Q  P  K  G  K  A  L  H  K  A  A  N
1426  GTAAGTTATGATTACTTCAACGTCGAGGAAGTTCTTGACATGATAATTGTGGAATTGAGTGCTGATGTAAAAATG
235    V  S  Y  D  Y  F  N  V  E  E  V  L  D  M  I  I  V  E  L  S  A  D  V  K  M
1501  GAAGCACATGAAGAGTACCAAGATGGTGATGATGGGTTTAGTCTTTTCTCATATTAGGGTTTTAGCTATGAGGGT
261    E  A  H  E  E  Y  Q  D  G  D  D  G  F  S  L  F  S  Y
```

(c)

```
1     AAGAAAACAGGCGACAAGATTGCGAACGCTAGATATCTGGACCCGATCCGGATCGGGTCGGCCATGACGGTAGAT
1                                                                  M  T  V  D
76    CGGAAGCACGCCGAGGCGGCGGCGGCGGCGCCCTTCGAGATCCCGGCGCTCCAGCCTGGAAGGAAAAAGCGACCT
5      R  K  H  A  E  A  A  A  A  A  P  F  E  I  P  A  L  Q  P  G  R  K  K  R  P
151   CGGAGATCACGTGATGGGCCTAATTCAGTCTCTGAAACGATCAGGCGATGGAAAGAAGTGAACCAACAACTGGAG
30     R  R  S  R  D  G  P  N  S  V  S  E  T  I  R  R  W  K  E  V  N  Q  Q  L  E
226   CATGATCCACAGGGTGCAAAGAGGGCGAGGAAGCCACCTGCAAAGGGTTCGAAGAAGGGCTGTATGCTGGGGAAA
55     H  D  P  Q  G  A  K  R  A  R  K  P  P  A  K  G  S  K  K  G  C  M  L  G  K
301   GGAGGACCTGAGAATACACAATGTGGATTCCGTGGTGTAAGGCAACGTACTTGGGGGAAGTGGGTTGCTGAAATT
80     G  G  P  E  N  T  Q  C  G  F  R  G  V  R  Q  R  T  W  G  K  W  V  A  E  I
376   CGGGAGCCAAATCGGGTGAGCAGGCTCTGGCTGGGAACGTTCCCCACTGCTGAGGATGCTGCCCGTGCTTATGAC
105    R  E  P  N  R  V  S  R  L  W  L  G  T  F  P  T  A  E  D  A  A  R  A  Y  D
451   GAGGCAGCCAGAGCAATGTATGGCGCACTGGCTCGTACCAACTTCCCTGTGCATCCTGCACAAGCTCCTGCTGTG
130    E  A  A  R  A  M  Y  G  A  L  A  R  T  N  F  P  V  H  P  A  Q  A  P  A  V
526   GCTGTACCAGCGGCAATCGAAGGTGTTGTACGTGGTGCTTCAGCATCATGCGAGTCTACTACAACGTCCACCAAC
155    A  V  P  A  A  I  E  G  V  V  R  G  A  S  A  S  C  E  S  T  T  T  S  T  N
601   CACTCAGATGTTGCTTCTTCCTTGCCGAGACAAGCTCAAGCTCCTGAGATTTACTCCCAGCCAGATGCGCTTGAG
180    H  S  D  V  A  S  S  L  P  R  Q  A  Q  A  P  E  I  Y  S  Q  P  D  A  L  E
676   TCCACAGAATCAGTTGTGCTGGAGTCTGTCGAGCATTACAGCCATCAAGACACTGTTCCTGACGCTGGCTCAAGC
205    S  T  E  S  V  V  L  E  S  V  E  H  Y  S  H  Q  D  T  V  P  D  A  G  S  S
751   ATTTCAAGGAGCACATCCGAAGAGGATGTGTTCGAGCCATTGGAGCCTATTTCCAGTTTGCCGGATGGAGAAGCA
230    I  S  R  S  T  S  E  E  D  V  F  E  P  L  E  P  I  S  S  L  P  D  G  E  A
826   GACGGTTTTGATATAGAAGAATTATTGAGATTGATGGAAGCCGACCCAATTGAAGTCGAGCTGGTCACTGGGGGC
255    D  G  F  D  I  E  E  L  L  R  L  M  E  A  D  P  I  E  V  E  L  V  T  G  G
901   TCCTGGAATGGTGGAGCCAACACTGGCGTGGAGATGGGCCAGCAGGAACCTCTCTACCTGGATGGCTTGGACCAA
280    S  W  N  G  G  A  N  T  G  V  E  M  G  Q  Q  E  P  L  Y  L  D  G  L  D  Q
976   GGCATGCTGGAGGGCATGCTGCAGTCTGATTATCCTTACCCAATGTGGATATCAGAGGATCGGGCCATGCACAAC
305    G  M  L  E  G  M  L  Q  S  D  Y  P  Y  P  M  W  I  S  E  D  R  A  M  H  N
1051  TCTGCCTTCCATGATGCTGAGATGAGCGAGTTCTTCGAAGGGTTGTGATCCCCTACCGGCG
330    S  A  F  H  D  A  E  M  S  E  F  F  E  G  L
```

Figure S1 The sequences of wheat *DREB* genes. (a), (b) and (c) represent the nucleotide sequence and amino acid sequence of *DREB2*, *DREB6* and *Wdreb2*, respectively. The box and underline denote nuclear localization signal and AP2/EREBP domain, respectively.

(a)

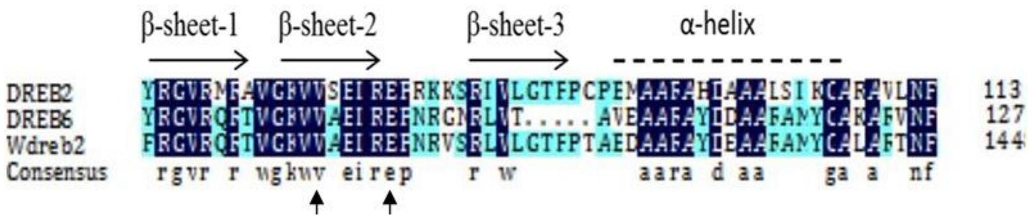

(b)

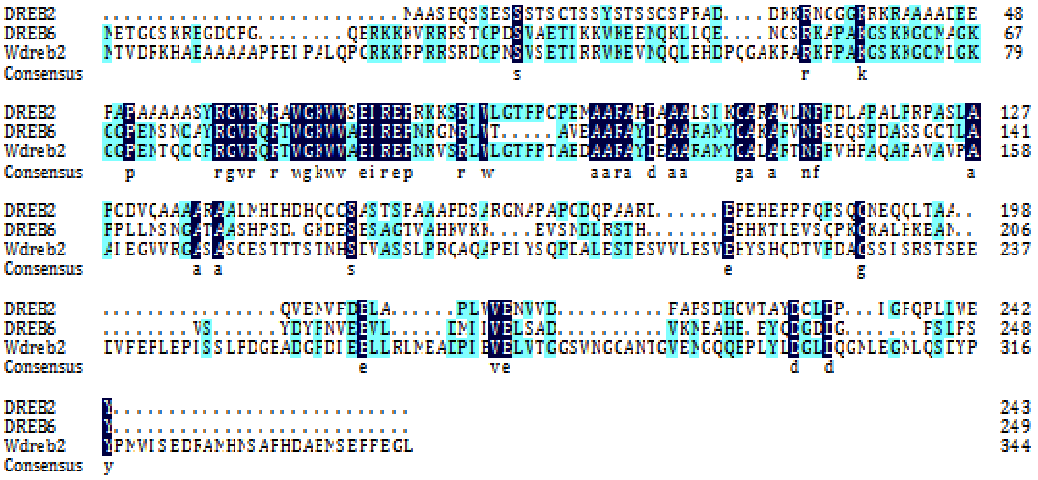

Figure S2 Alignments of wheat DREBs. (a) and (b) represent the alignments of AP2/EREBP domain and amino acid sequence of *DREB2*, *DREB6* and *Wdreb2*. The similar and consistent amino acids are indicated in blue or dark blue, respectively. β-sheet and α-helix are indicated with long arrow and dotted line, the conserved residues at 14 th and 19 th are marked with short arrow.

(a)

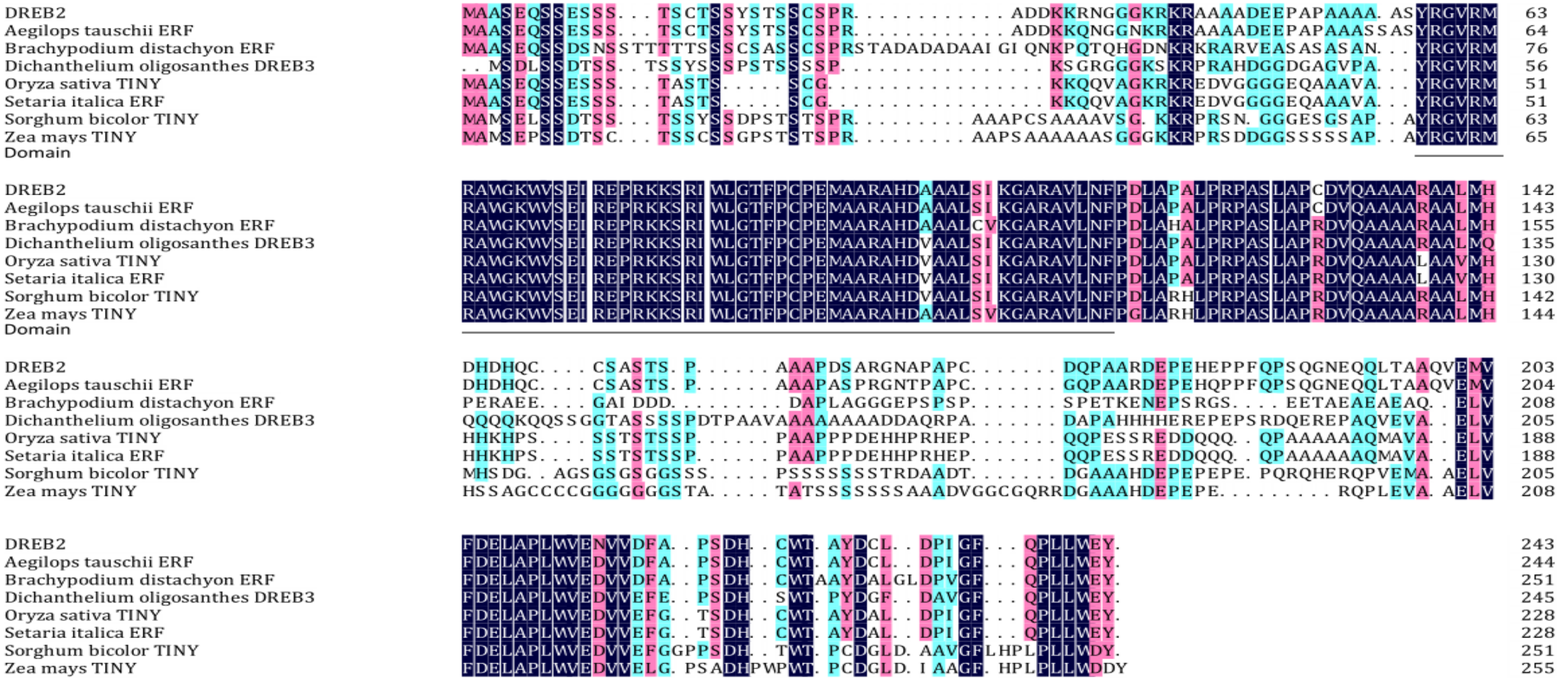
DREB2 MAASEQSSESSS...TSCTSSYSTSSCSPR.............ADDKKRNGGGKRKRAAAADEEPAPAAAA.ASYRGVRM 63
Aegilops tauschii ERF MAASEQSSESSS...TSCTSSYSTSSCSPR.............ADDKKQNGGNKRKRAAAADEEPAPAAASSASYRGVRM 64
Brachypodium distachyon ERF MAASEQSSDSNSSTTTTTSSSCSASSCSPRSTADADADAAI GI QNKPQTQHGDNKRKRARVEASASASAN...YRGVRM 76
Dichanthelium oligosanthes DREB3 ..MSDLSSDTSS..TSSYSSSPSTSSSSP.................KSGRGGGKSKRPRAHDGGDGAGVPA...YRGVRM 56
Oryza sativa TINY MAASEQSSESSS...TASTS.....SCG.................KKQQVAGKRKREDVGGGGEQAAAVA...YRGVRM 51
Setaria italica ERF MAASEQSSESSS...TASTS.....SCG.................KKQQVAGKRKREDVGGGGEQAAAVA...YRGVRM 51
Sorghum bicolor TINY MAMSELSSDTSS...TSSYSSDPSTSTSPR.........AAAPCSAAAAVSG.KKRPRSN.GGGESGSAP..AYRGVRM 63
Zea mays TINY MAMSEPSSDTSC...TSSCSSGPSTSTSPR.........AAPSAAAAAASGGGKKRPRSDDGGSSSSSAP..AYRGVRM 65
Domain
DREB2 RAWGKWVSEIREPRKKSRIWLGTFPCPEMAARAHDAAALSIKGARAVLNFPDLAPALPRPASLAPCDVQAAAARAALMH 142
Aegilops tauschii ERF RAWGKWVSEIREPRKKSRIWLGTFPCPEMAARAHDAAALSIKGARAVLNFPDLAPALPRPASLAPCDVQAAAARAALMH 143
Brachypodium distachyon ERF RAWGKWVSEIREPRKKSRIWLGTFPCPEMAARAHDAAALCVKGARAVLNFPDLAHALPRPASLAPRDVQAAAARAALMH 155
Dichanthelium oligosanthes DREB3 RAWGKWVSEIREPRKKSRIWLGTFPCPEMAARAHDVAALSIKGARAVLNFPDLAPALPRPASLAPRDVQAAAARAALMQ 135
Oryza sativa TINY RAWGKWVSEIREPRKKSRIWLGTFPCPEMAARAHDVAALSIKGARAVLNFPDLAPALPRPASLAPRDVQAAAALAAVMH 130
Setaria italica ERF RAWGKWVSEIREPRKKSRIWLGTFPCPEMAARAHDVAALSIKGARAVLNFPDLAPALPRPASLAPRDVQAAAALAAVMH 130
Sorghum bicolor TINY RAWGKWVSEIREPRKKSRIWLGTFPCPEMAARAHDVAALSIKGARAVLNFPDLARHLPRPASLAPRDVQAAAARAALMH 142
Zea mays TINY RAWGKWVSEIREPRKKSRIWLGTFPCPEMAARAHDAAALSVKGARAVLNFPGLARHLPRPASLAPRDVQAAAARAALMH 144
Domain
DREB2 DHDHQC....CSASTS.P......AAAPDSARGNAPAPC.......DQPAARDEPEHEPPFQPSQGNEQQLTAAQVEMV 203
Aegilops tauschii ERF DHDHQC....CSASTS.P......AAAPASPRGNTPAPC.......GQPAARDEPEHQPPFQPSQGNEQQLTAAQVEMV 204
Brachypodium distachyon ERF PERAEE....GAI DDD.........DAPLAGGGEPSPSP.......SPETKENEPSRGS....EETAEAEAEAQ..ELV 208
Dichanthelium oligosanthes DREB3 QQQQKQQSSGGTASSSSPDTPAAVAAAAAAAADDAQRPA.......DAPAHHHHEREPEPSRDQEREPAQVEVA..ELV 205
Oryza sativa TINY HHKHPS....SSTSTSSP......PAAPPPDEHHPRHEP.......QQPESSREDDQQQ..QPAAAAAAQMAVA..ELV 188
Setaria italica ERF HHKHPS....SSTSTSSP......PAAPPPDEHHPRHEP.......QQPESSREDDQQQ..QPAAAAAAQMAVA..ELV 188
Sorghum bicolor TINY MHSDG..AGSGSGSGGSSS.....PSSSSSSSTRDAADT.......DGAAAHDEPEPEPE.PQRQHERQPVEMA.AELV 205
Zea mays TINY HSSAGCCCCGGGGGGGSTA.....TATSSSSSSSAAADVGGCGQRRDGAAAHDEPEPE.........RQPLEVA.AELV 208
DREB2 FDELAPLWVENVVDFA..PSDH..CWT.AYDCL..DPI GF...QPLLWEY. 243
Aegilops tauschii ERF FDELAPLWVEDVVDFA..PSDH..CWT.AYDCL..DPI GF...QPLLWEY. 244
Brachypodium distachyon ERF FDELAPLWVEDVVDFA..PSDH..CWTAAYDALGLDPVGF...QPLLWEY. 251
Dichanthelium oligosanthes DREB3 FDELAPLWVEDVVEFE..PSDH..SWT.PYDGF..DAVGF...QPLLWEY. 245
Oryza sativa TINY FDELAPLWVEDVVEFG..TSDH..CWT.AYDAL..DPI GF...QPLLWEY. 228
Setaria italica ERF FDELAPLWVEDVVEFG..TSDH..CWT.AYDAL..DPI GF...QPLLWEY. 228
Sorghum bicolor TINY FDELAPLWVEDVVEFGGPPSDH..TWT.PCDGLD.AAVGFLHPLPLLWDY. 251
Zea mays TINY FDELAPLWVEDVVELG.PSADHPWPWT.PCDGLD.I AAGF.HPLPLLWDDY 255

(b)

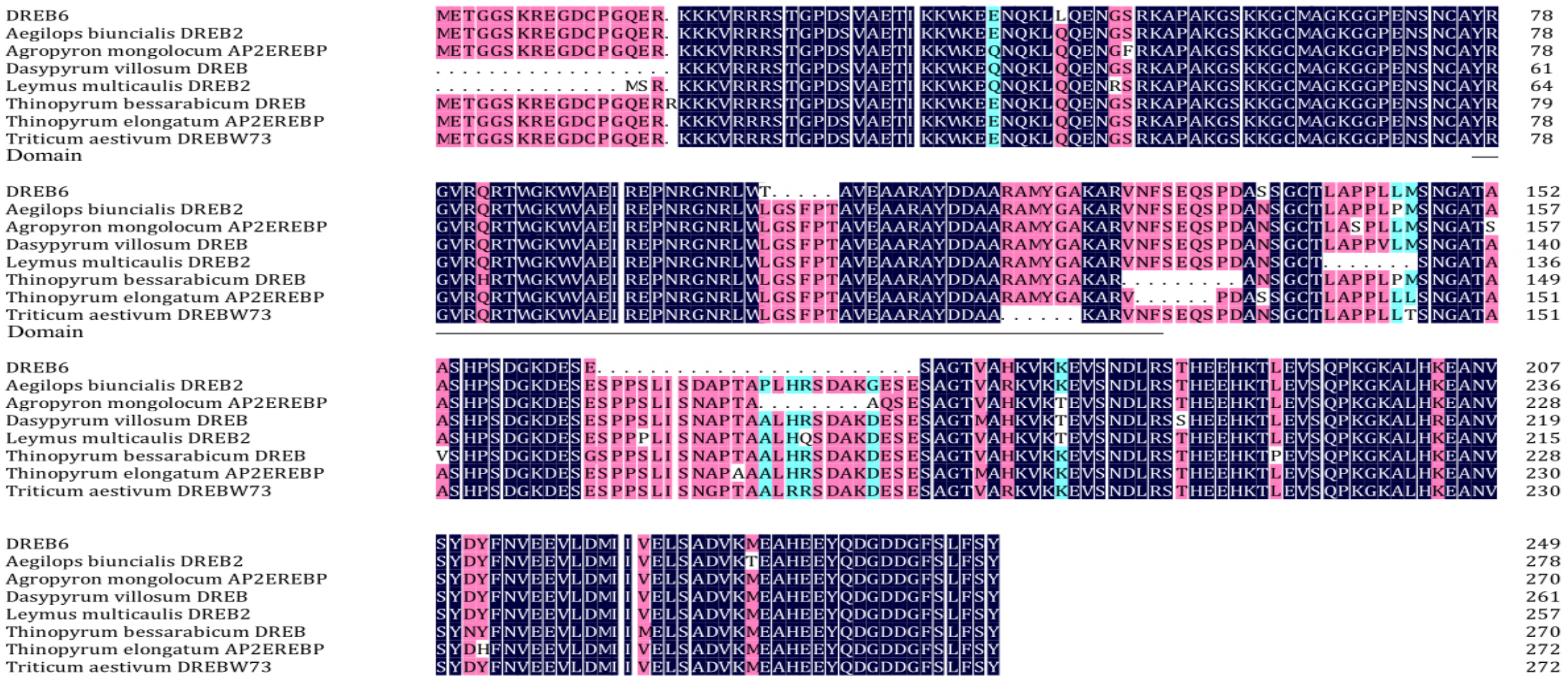
DREB6
Aegilops biuncialis DREB2
Agropyron mongolocum AP2EREBP
Dasypyrum villosum DREB
Leymus multicaulis DREB2
Thinopyrum bessarabicum DREB
Thinopyrum elongatum AP2EREBP
Triticum aestivum DREBW73
Domain

(c)

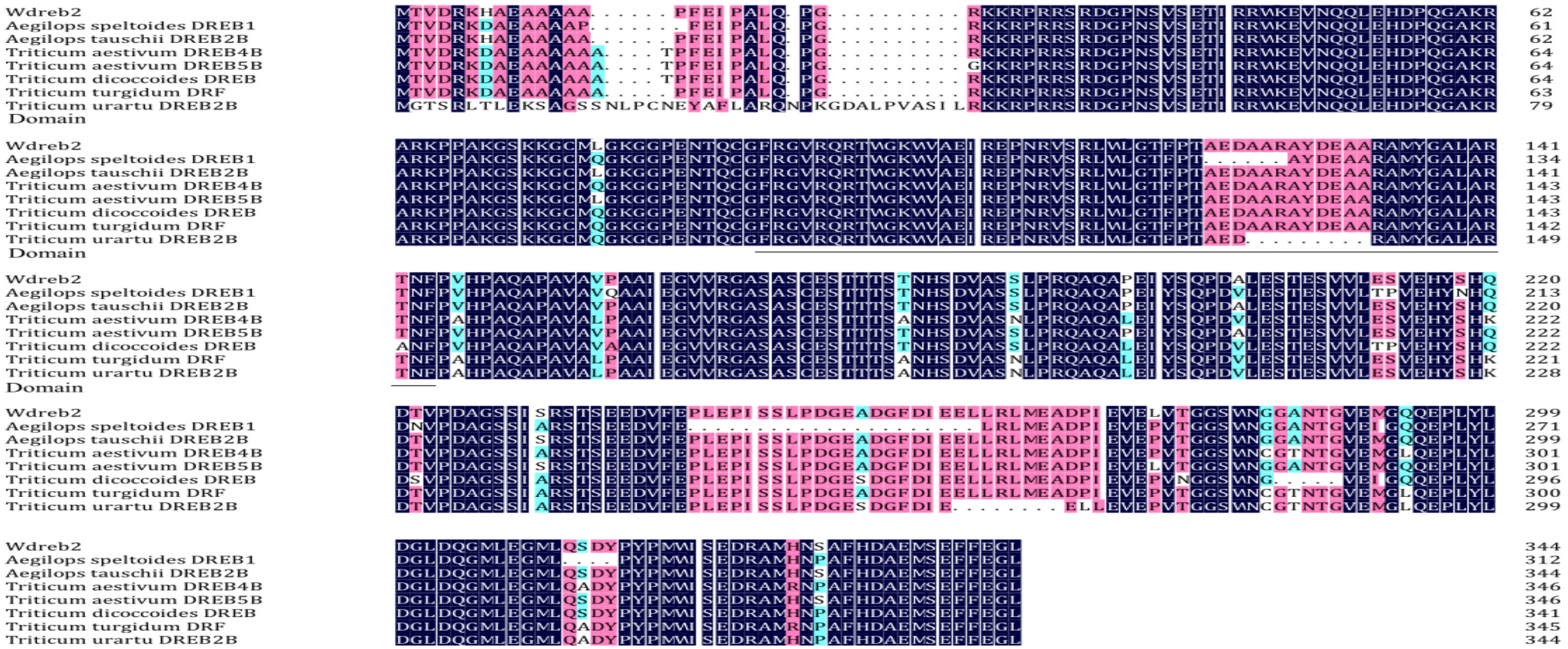

Figure S3 Multiple alignment of wheat DREBs and its homologous sequences. (a), (b) and (c) are the alignment on some homologous sequences of wheat DREB2, DREB6 and Wdreb2, respectively. The underline denotes the AP2/EREBP domain.

(a)

GGTCGGCAGC ACTACTATGC TCGCCTCATT GCCCTGCAAA AGCCTGAACC GCCACCCACC GATGTGATAT CGCTTCCCCG

GCCATCCCTC TAATCCTCCT TCCCCCAACC CAAACAATAA ACCCTCTTAT TCCCTCCCGT TTTCAACGCC GTGCTCGTGG
CAAT-box

CTGCATTGAA ATCAATTGAT GCATCATTGT TTTTAGGTGT ATGAGCTTTA ACCCAATTTT TTTACTATTT ATTATTAGGG
AT-rich element CAAT-box TATA-box GT1-motif

TCTGAGCAAT AACTATCTCT AAGTTCACCA CTAACCATCC TAACATGAGA CTTATACAAG TACAAAAGAC ACGACAAGAT
CAAT-box

CTTGATCAGA TGGTTATCAA TATCAAATGC AAAACATTTT TGGAGCCGGC CCTTTGCTGA AGATTGTTGG AACTTTGTTT

ATCCCTGAAG AAACATAAAT CTGTTGCTCT TGAGGCCAGA TCCAAAGGAA AAGTCAAGGG TGCCTTTCTT TATGGAAATT
GARE-motif

ATTGTACTTG CTTCCTGGGC CATCTGGATT TCCAAAAGCA ATAAGATATT CATATGAGGC GTGCAGTTTA ATGAGATGGA
CAAT-box

AATAAATTTG CCCCCAAGAG CTCACCCTGC TGAAATTTAG GATTAAGAGG AGACATGAAG TTGTTTTTGT CTTCAGGTCC

GACTCCTTTT GTAAACTTTA ATCTGTAATC TTTCTTTCCT TCCTTTTTTT TTCTTCCTTA TTAGGCTTAT CTGTAGTCAT
P-box

TTGTTGATAT TGTTTAAATT CTTTCATTAA GAGTGAATTC TATTATTTTT ACCTCTTAGT GTCACGTTTG AGACATCAAC
TATA-box G-box MBS

TGCTATATTT AACTTCCCGC AAAAAAAAGA ACTGCTATAT TTAACAGGTT TTCACATATT TACCCAATTT AAGCAAACTG
CAAT-box

GTGCGACAAA TTATCCTATT TTTTTCTCTT TAAATGTTGA TTGTACATGC CATTTTGGCT GTTTTTTTTA CTTTTTATTT
TATA-box TATA-box

GCTCTTGATT TGTCAAGGGA TGGGTCCTAC CTGCCAGTTG GGGAGCAAGA AATATGAGTG AAAAATTGGA GTACGGCTGT

AGTTTGAACC CGAAATCTCC CCCCGCAGAC AGGTAAGACT CATCCCCCGC CGGACCGAGA GCAAACCAGA CAAAACCAAA
LTR GC-motif TCA-motif GC-motif ARE ARE

CAGTAAAACA AACCAGCCAA TATGGCATGC ACAGTCAACA CTGATTCAGA AAATAAATAA ATAAATGGGG ATAATTTGTG
ARE

GCACAATTTT GCTTACATTG GGTAAAAGTA CATGGAAAAA CTACCAAATG GGACAATTCT TGTCACAAAC GTGAAACGTG
CAAT-box CAAT-box ABRE ABRE

GGGTACAAAT AGAATTAACC CTGATGTAAT TCCATTTGTA CATTTTCCTC TGACAATTAT TGAAAAAGTA GAGTTACAGT
GT1-motif CAAT-box

AGGGTTTTAC CCTACTATTT TTCTCAAAAA AAAAGAATTC ACTCTTTAAT TAATTTATAA AAATTGTAGT GGGGTATTAT
TATA-box

CCTACCGTTG CCCTTCAAAA GAAAAATGTC GAGTGAGAAA TAATTATTTC TAATTCGACC GTGCTATAGA CGTTTTTATT
CCAAT-box TATA-box

TCTAAGACAA CATATACTGC GTTCTGAAAT GTCAGGGAGT CCAAGTGAAT CGTTGGGAGG AATTGAATGT CTAGGTGTAG

CAGGGCATGG AGTCAGGAGA GGGAGGCATG CAGCCCCGCC CGGTCCTGTC CGACCCTGCG AGAGACCATG CGACCCTAAT

TATTGATGAT CATGGCGGAG TCAGCTGTCG TTCATGGGTT CCTATCCCAC GCCACGTTGC CTCCACTCTC CTCTACATAT
TGA-element GATA-motif TATC-box G-box

ACAGCACCAC CTCCCTCTTC CTCCTCGCTC TACCCCTCTC CTTGCTCGCA GTAACTCGAG TCCTACTCCC TGCACGGTCC

TCCCATTACC ACGAGCGAGC GCGTGTCATC TCATACGGGC ACGCGGGGCT ACGTGGTGCT CTCTGCGCGC ACATG
Sp1 Skn-1-motif ABRE

(b)

TTCCCCACTA GAAGCATAAC TTATGCATCT GCACTATGAG TGGACGGGGA AATAGATGCT TAGTGGTGGC AGTGTTTTGA

A-box

ACCTCTTTAG CGGAATATGT TGTTGTCATC GTTTTGCTTC TGTCGTTGCT TCAGTTACAA ATGGTTCTCT TTGAAGGCCA

TGA-element

GACATATCCA TCTGACACAA ACCGTGTGAT GATATAACGA AGTTCCAATT CACGATGTGA AGTGCTCATG TGATGTATTT

CAAT-box

CTATATTAGG TGATCCCACA TGAGCATCGA GAAAACTCAA GATCTTAAAA TGCCAAAAAA CGGTCATATC TTTTATGTCC

TATA-box MBSI TATA-box

TCGCAAGGAG ATGCCTAATT TCATCATGAA CCCACTCTGG CCATGGCACC AATACTAATC ATGCCAAGAT AGATCGTCCG

CAAT-box

AACACTATAC CTACGTACAC TTACATCTAC CATGAGACCT CAAACAAGAG CCTCACGTGT CATGCACCAC TGGATATCAG

G-box ABRE

ACGAAAGCCT TAGTGCATAT CTCTTGACAA CAAGACAACT GATGCCGATG TTGTGTCCAT TCCCAATGTC ACCTTTTTCC

MBS CAAT-box

GCCCCGTCTC CTCACGACGA GAAGAGGATA CAGGTCTGTT TGGATGACGC TGACAACGCC CCCGCAAAAA ATGGATGTGC

SP1 GARE-motif CGTCA-motif GC-motif

CAATAATTTC AAGGAGGAAT TCAGATCCTT GGTTTCGCCA ACACGTAGTG TGTAGAAATT GAACCGACGA TCGAAAAGTT

CAAT-box ACE ABRE

GGTGGAGGTT CAAACGTTCG CCCATGCTCC CGGTCCATGC TGAAAAAATG GTGTGGAGAG ACCTGATTTT CACAAGCCCA

AuxRR-core

CTTTGACCAT GGTACCAGCC ATGCCAAGAT AGATCGTCCG AACACTATAC CCATGCAGTC GAGCTACATG CACTTGTCTG

W-box

GTTCGATTGT TCAGTGGCTC TTTCTAAGAG GCGGGGAATG TAAATAGAAA ACATCCCACG AGGAAGGAAT CAAGTGGGAC

CGAGAGATCA TTGCTCATTC GGTGGTTGCC CTCCCCCCCT AATAAGTAAT CCCTCTGCAA AGTAATATAA GAGCGTTTAG

ATCACTATAA AAGGGAGTAC CTATCAAGAA TTGGGCCAGT TAGTATTTTC ATAGAAAAAA AATACCCTTT CCAGGTTTAA

Box-E MBS

TGATCAAACA TCATCTAGAT AATGACAACG TACCGAGTAG GCCCTACATC TATCGGGGAG CTCACATCCT CACAAGAGCG

Skn-1 motif ABRE

AGGCACATAC TCTCGTGTCT ATATCACGAG ATCTCGAACA AGAGCCATGT GTGACGTCTA CCGTGGGGTC TCAAACGGCA

TGACG-motif

ACCCCGCGTG TCTATCTGTT GACATCAAGA CAACTGCCAG CGATGCTCTT GTCCATTTTC AACGTCACCT TTTCCCGCCC

MBS CGTCA-motif

GCCTTCCCTC GGCGGGAAGA AAGATACGTG TCTATTTGGA GCGGCGCCCA CGCCAACCCC ACAAACTCGG GCGCGCCAAT

ABRE

AATTTGACGA AGGAATTTAC ATCATCGGTT TCACCGACAC ACGTGGCGTA AAAGCTGAAC CAACTACTGA AAAGTCGGCG

ABRE

GAGGTCCACG TGCACCGATG CCGAAAATTG ATAGGGCAGC TCCAGACAGC ATTCCAAACA AACCCTACAT AGTGGCAACA

LTR

TCACACGAAA AATGTTGAGA TCAGCCTCTC CCGCTCCCTC TTTTCATCAA AAGGAAAACG GCGGAAACGA AATTTGAAAT

ERE

CGGCGCCCGA CGGTGATACT CCTATATCGC AGCGAATCCA GGGGGGATCG AGTCGGCGTG TCCGTCCGTT TGGAAGGCTG

CRT/DRE SP1 A-box

CCTGCAAGCA GGTTACGACT TGCCTTTACT GG

(c)

ATCCGTTCAC ATGGACAGCG GGCGAGCCTT AGGTTTGCTC GAAGCATTAT GCCAGCGTTT TCATCCGTTT TAATTCAAAC

MRE

AGACACACTA GAATCAAAAT GCATCGACAC GTTGGAGTTA CCCTTACAAT GGAGAAGAGA AGACCACGCC GGCCACCACT

ABRE CAAT-box

GATCCATGCA ACCAAAGGCG TCTCACGCCG TCTCAAGAAT AAAAAATGCC TCACGATCCA CCGTGTCATC CCGACTGCCC

O2-site

ACGTGTCGTC TCCTATATCG ACTCAGCCCC CAGCCACTGG CCACATGTAG ACGTACACGT GGAACCCACC CAACAGCAGC

ABRE TATA-box CAT-box ABRE

ACGTCCAATC CACCCCGAAA GAAAAGCACC GGTAGCACTC GAAAGCGTAA AGGAGAAGCC GAGTTTCCTT CGGCACCAAG

CAAT-box LTR

TCTCGGGAAG TTTCCCCGCA CCACGGCACA GCTGACGTCA CGCGTCCGCA GCCTCACCCC CACGCACCAG GCCCACGCGG

TGACG-motif

ACCGCGTCGC TTACATCCGG GCCCCACGCG CCAACGCCCC GCACGCGAGT GACCTAGATA ATTCCGTGAG TCCGGAGCAC

GGAGCACCCC GATCCGGGCT ATAATAGGCG CCCCGGCGTG AGCCCCTGGG GTTTCCGACT TTCTTTCTCT CTTCCTCCAA

TATA-box

CTCTCTATCC AAGTCGACGC AGCGAAGAAA CAGGCGACAA GATTGCGAAC GCTAGATATC TGGACCCGAT CCGGATCGGG

CAAT-box

TCGGCCATC

Figure S4 Promoter regions of wheat *DREB* genes. (a), (b) and (c) represent the promoter sequences and some *cis*-acting elements of *DREB2*, *DREB6* and *Wdreb2*, respectively.

(a)

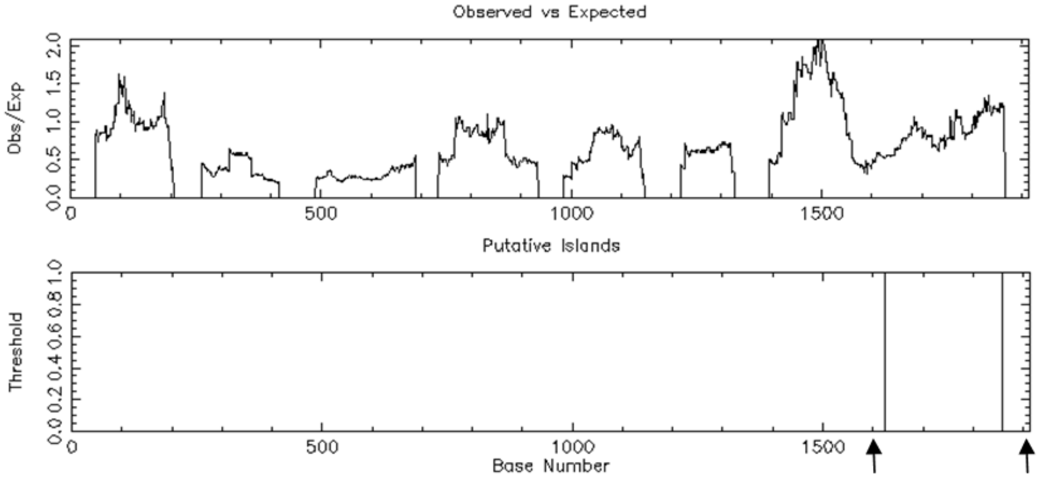

(b)

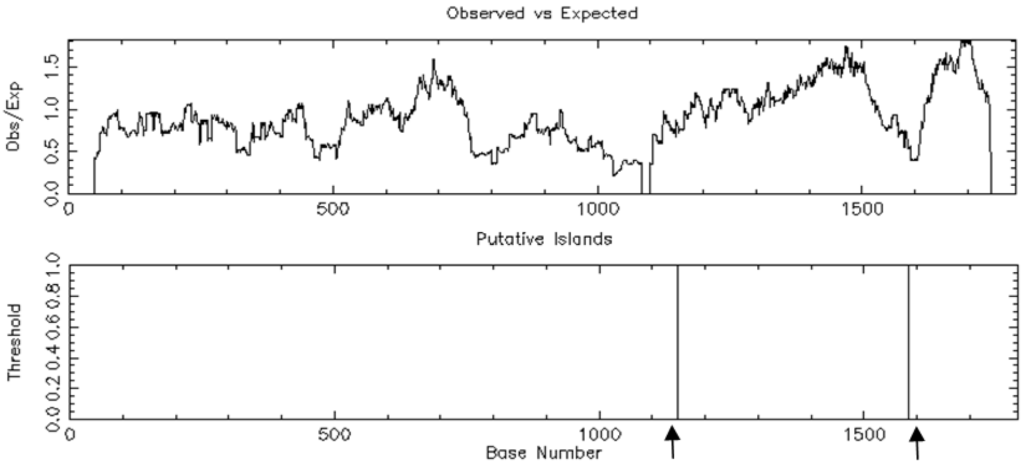

(c)

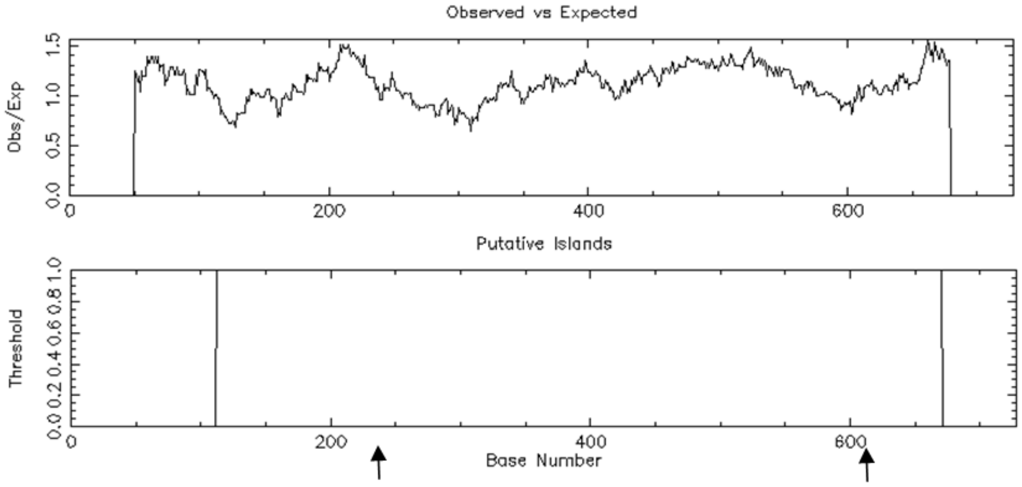

Figure S5 Prediction of CpG islands in the promoter regions of wheat *DREB* genes. (a), (b) and (c) represent the location of putative CpG islands in the promoter regions of *DREB2*, *DREB6* and *Wdreb2*, respectively. The CpG island regions between two arrows below each diagram were examined using BSP, respectively.

(a)

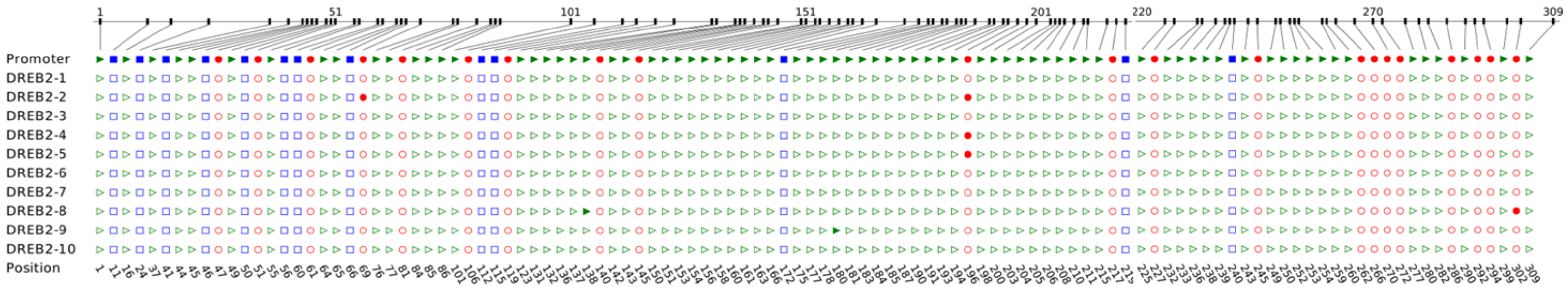

(b)

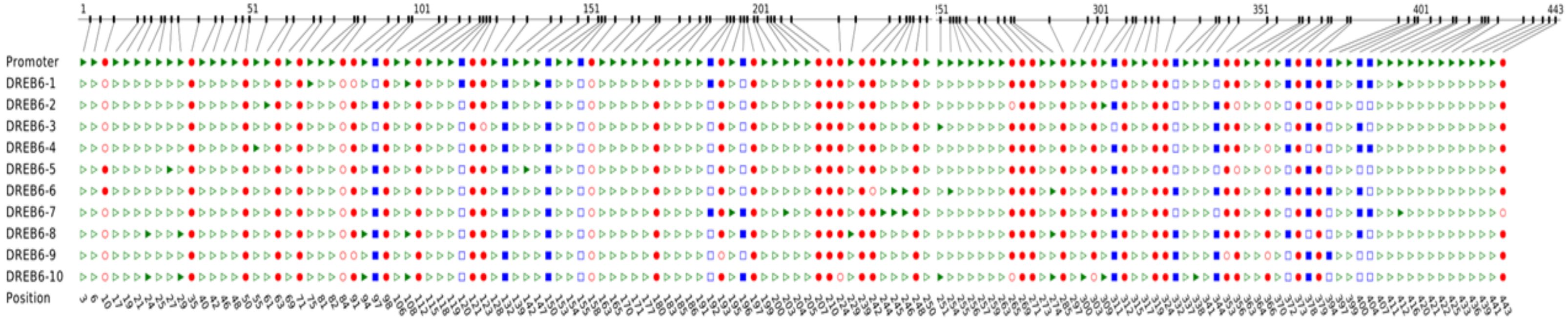

(c)

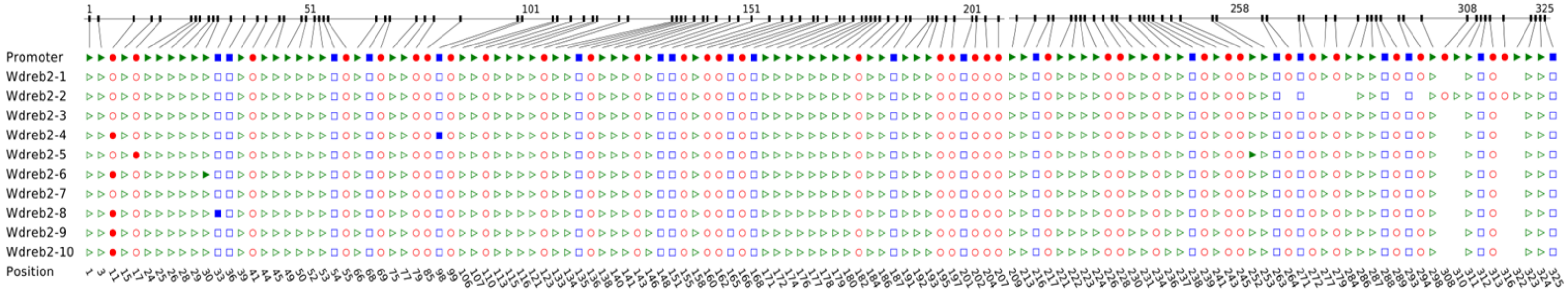

Figure S6 Bisulfite sequencing analysis on the promoter regions of wheat *DREB* genes using CyMATE. (a), (b) and (c) indicate methylation sites in the promoter regions of *DREB2*, *DREB6* and W*dreb2* in wheat leaves. Circles, squares and triangles represent CG, CHG or CHH, filled and hollow circles denote methylated and unmethylated cytosine. Each row represents the sequencing result of one positive clone. The length of CpG island sequence is visualized above each diagram and the potential methylation sites are displayed below.

Table S1 Homologous amino acid sequences of wheat DREBs by BLASP

	Protein	Accession Number	Identity
DREB2	*Aegilops tauschii* ERF	XP-020183719.1	95%
	Setaria italica ERF	XP-004968548.2	66%
	Oryza sativa TINY	XP-015644400.1	64%
	Brachypodium distachyon ERF	XP-010233006.1	64%
	Dichanthelium oligosanthes DREB3	OEL19602.1	61%
	Sorghum bicolor TINY	XP-002454993.1	61%
	Zea mays TINY	XP-020398183.1	59%
DREB6	*Thinopyrum elongatum* AP2/EREBP	AEI98920.1	98%
	Triticum aestivum DREBW73	AAY44604.1	98%
	Agropyron mongolocum AP2/EREBP	AJD80690.1	94%
	Aegilops biuncialis DREB2	CBX87024.1	97%
	Leymus multicaulis DREB2	AFO12475.1	97%
	Thinopyrum bessarabicum DREB	AIY22662.1	96%
	Dasypyrum villosum DREB	AIY22669.1	97%
Wdreb2	*Aegilops tauschii* DREB2B	XP-020156298.1	99%
	Triticum aestivum DREB5B	AAX13287.1	99%
	Aegilops speltoides DREB1	AC035588.1	96%
	Triticum turgidum DRF	AFO10996.1	95%
	Triticum aestivum DREB4B	AAX13283.1	94%
	Triticum dicoccoides DREB	ADM93284.1	93%
	Triticum urartu DREB2B	EMS45041.1	93%

The homologous sequences of wheat DREB2, DREB6 and Wdreb2 were retrieved with BlastP algorithm, which are mainly from some species in Gramineae. The homologous sequences having higher identity with wheat DREBs were selected from some Genera in Gramineae, and were further analyzed.

Table S2 The *cis*-acting elements in the promoter of *DREB2*

Element	Sequence	Function	Number
TATA-box	TATAWAW	Core promoter element around -30 of transcription start	13
CAAT-box	CAAT	Common cis-acting element in promoter and enhancer regions	34
ABRE	CGTACGTGCA	*Cis*-acting element involved in the abscisic acid responsiveness	5
ACE	AAAACGTTTA	*Cis*-acting element involved in light responsiveness	1
ARE	TGGTTT	*Cis*-acting regulatory element essential for the anaerobic induction	3
MBS	CAACTG	MYB binding site involved in drought-inducibility	2
LTR	CCGAAA	*Cis*-acting element involved in low-temperature responsiveness	1
G-box	(T/C)ACGT(C/G/T)	*Cis*-acting regulatory element involved in light responsiveness	6
C-repeat/DRE	TGGCCGAC	Regulatory element involved in cold- and dehydration-responsiveness	1
EIER	TTCGACC	Elicitor responsive element	1
TGACG-motif	TGACG	*Cis*-acting regulatory element involved in the MeJA-responsiveness	1
Skn-1-motif	GTCAT	*Cis*-acting regulatory element required for endosperm expression	1
SP1	CC(G/A)CCC	Light responsive element	4
GARE-motif	TCTGTTG	Gibberellin responsive element	1
TCA-element	TCAGAAGAGG	*Cis*-acting element involved in salicylic acid responsiveness	2
TGA-element	AACGAC	Auxin responsive element	1
OCT	CGCGGATC	*Cis*-acting regulatory element related to meristem specific activation	1
TATC-box	TATCCCA	*Cis*-acting element involved in gibberellins responsiveness	1
Circadian	CAANNNNATC	*Cis*-acting regulatory element involved in circadian control	1
MNF	GTGCCC(A/T)	Light responsive element	1
HD-Zip	CAAT(A/T)ATTG	Element involved in differentiation of the palisade mesophyll cells	1
GT1-motif	GGTTAAT	Light responsive element	2
GC-motif	CCCCCG	Enhancer-like element involved in anoxic specific inducibility	2
TATC-box	TATCCCA	*Cis*-acting element involved in gibberellins responsiveness	1
P-box	CCTTTTG	Gibberellins responsive element	1

Table S3 The *cis*-acting elements in the promoter of *DREB6*

Element	Sequence	Function	Number
TATA-box	TATAWAW	Core promoter element around -30 of transcription start	10
CAAT-box	CAAT	Common *cis*-acting element in promoter and enhancer regions	19
ABRE	CGTACGTGCA	*Cis*-acting element involved in the abscisic acid responsiveness	10
ACE	AAAACGTTTA	*Cis*-acting element involved in light responsiveness	2
ARE	TGGTTT1	*Cis*-acting regulatory element essential for the anaerobic induction	1
MBS	CAACTG	MYB binding site involved in drought-inducibility	5
LTR	CCGAAA	*Cis*-acting element involved in low-temperature responsiveness	1
AuxRR-core	GGTCCAT	*Cis*-acting regulatory element involved in auxin responsiveness	1
TGA-element	AACGAC	Auxin responsive element	1
TGACG-motif	TGACG	*Cis*-acting regulatory element involved in the MeJA-responsiveness	4
CGTCA-motif	CGTCA	*Cis*-acting regulatory element involved in the MeJA-responsiveness	3
CAT-box	GCCACT	*Cis*-acting regulatory element related to meristem expression	2
G-box	(C/T)ACG(T/A)G	*Cis*-acting regulatory element involved in light responsiveness	11
MBSI	aaAaaC(G/C)GTTA	MYB binding site involved in flavonoid biosynthetic genes regulation	1
A-box	CCGTCC	*Cis*-acting regulatory element	2
GARE-motif	AAACAGA	Gibberellin responsive element	2
P-box	CCTTTTG	Gibberellin responsive element	1
Circadian	CAANNNNATC	*Cis*-acting regulatory element involved in circadian control	1
DRE/CRT	RCCGAC	Drought, high salt and cold-responsive element	1
ERE	ATTTCAAA	Ethylene-responsive element	1
SP1	GGGCGG	Light responsive element	6
Box I	TTTCAAA	Light responsive element	1
GC-motif	CCCCCG	Enhancer-like element involved in anoxic specific inducibility	1
W-box	TTGACC	Fungal elicitor responsive element	1

Table S4 The *cis*-acting elements in the promoter of *Wdreb2*

Element	Sequence	Function	Number
5UTRpy-rich stretch	TTTCTTCTCT	*Cis*-acting element conferring high transcription levels	1
TATA-box	TATAWAW	Core promoter element around -30 of transcription start	5
CAAT-box	CAAT	Common *cis*-acting element in promoter and enhancer regions	4
CAT-box	GCCACT	*Cis*-acting regulatory element related to meristem expression	1
ABRE	CGTACGTGCA	*Cis*-acting element involved in the abscisic acid responsiveness	8
ACE	AAAACGTTTA	*Cis*-acting element involved in light responsiveness	1
O2-site	GATGACATGG	*Cis*-acting regulatory element involved in zein metabolism	1
C-repeat/DRE	TGGCCGAC	Regulatory element involved in cold and dehydration responsiveness	1
C-box	CTGACGTCAG	*Cis*-acting regulatory element involved in light responsiveness	1
CE3	GACGCGTGTC	*Cis*-acting element involved in ABA and VP1 responsiveness	1
G-box	CACGT(G/C/T)	*Cis*-acting regulatory element involved in light responsiveness	7
MRE	AACCTAA	MYB binding site involved in light responsiveness	1
LTR	CCGAAA	*Cis*-acting element involved in low-temperature responsiveness	1
TGACG-motif	TGACG	*Cis*-acting regulatory element involved in the MeJA-responsiveness	2
GARE-motif	TCTGTTG	Gibberellin responsive element	1
SP1	CCGCCC	Light responsive element	2
as1	TGACGTCA	*Cis*-acting regulatory element involved in the root specificexpression	1
CGTCA-motif	CGTCA	*Cis*-acting regulatory element involved in the MeJA-responsiveness	2

Table S5 Correlation between promoter methylation and expression of *DREB* genes

Gene / Methylation type	Pearson's r		
	DREB2	*DREB6*	*Wdreb2*
CG	-0.952	-0.981	-0.986
CHG	-0.327	-0.950	-0.973
CHH	-0.509	0.656	-0.878

Pearson's r represents the relationship between expression levels of *DREB2, DREB6, Wdreb2* in wheat leaves and methylation rates at CG, CHG or CHH sites in their promoter regions under the treatment of 15% PEG_{6000} solution. According to the expression level and methylation rate when wheat seedlings incubated in PEG_{6000} solution for 12 h, Pearson's r analysis of *DREBs* were performed.

Table S6 Primers used in this study

Experiment	Primer	Sequence（5’ to 3’）
Gene cloning	*DREB2*-F	CTACTCCCTGCACGGTCCT
	DREB2-R	GCCAAGCCTAATACTCCCAC
	DREB6-F	CGTCCCTCTTCTCGCTCCA
	DREB6-R	CTAATATGAGAAAAGACTAAACCC
	Wdreb2-F	AAGAAAACAGGCGACAAGAT
	Wdreb2-R	ACGAAGCACAAAAAACTAGC
qRT-PCR	*β-Actin*-F	CCAAGGCGGAGTACGATGAGTCT
	β-Actin-R	TTCATACAGCAGGCAAGCACCAT
	DREB2-F	GCGTACAACACCTTGATTTCC
	DREB2-R	AAACTCAACTCACATCTAAGC
	DREB6-F	CGGCTGTGGCTTGGTTCATTC
	DREB6-R	CAGTTGCCCCATTAGACATC
	Wdreb2-F	AGATGTTGCTTCTTCCTTGCC
	Wdreb2-R	GATGTGCTCCTTGAAATGCTTG
Promoter cloning	*DREB2*-F	CCCAAGCTTGGTCGGCAGCACTACTATG
	DREB2-R	CATGCCATGGCATGTGCGCGCAGAGAGC
	DREB6-F	CCCAAGCTTTTCCCCACTAGAAGCATAAC
	DREB6-R	AACTGCAGCCAGTAAAGGCAAGTCGTAA
	Wdreb2-F	CCCAAGCTTATCCGTTCACATGGACAGC
	Wdreb2-R	AACTGCAGGATGGCCGACCCGATCCGGAT
BSP	*DREB2*-F	GTTTAGGTGTAGTAGGGTATGGAG
	DREB2-R	AAAAACACCACRTAACCC
	DREB6-1-F	TTTATATTTATYGGGGAGTTTATA
	DREB6-1-R	AAAACCRATAATATAAATTCCTTC
	DREB6-2-F	TGTTAGYGATGTTTTTGTTTAT
	DREB6-2-R	CAACATTTTTCRTATAATATTACCAC
	Wdreb2-F	GTTATATGTAGAYGTATAYGTGGA
	Wdreb2-R	AAAACCCCAAAAACTCAC
